# Optimization of a gluten‐free sponge cake formulation based on quinoa, oleaster, and pumpkin flour using mixture design methodology

**DOI:** 10.1002/fsn3.3977

**Published:** 2024-02-08

**Authors:** Mahshad Madadi, Sahar Roshanak, Fakhri Shahidi, Mohammad Javad Varidi

**Affiliations:** ^1^ Department of Food Science and Technology, Faculty of Agriculture Ferdowsi University of Mashhad Mashhad Khorasan Razavi Iran

**Keywords:** celiac, gluten‐free sponge cake, mixture design, oleaster, pumpkin, quinoa

## Abstract

Gluten‐free bakery products are the definitive solution for people with celiac disease and gluten sensitivity. In this study, the production of gluten‐free sponge cake was optimized using a mixture design methodology. Effects of the amount of ingredients, including quinoa (*Chenopodium quinoa*) (6–10 g), oleaster (*Eleagnus Angustifolia*) (1–2 g), and pumpkin powder (*Cucurbita moschata*) (1–4 g) on the physicochemical characteristics and sensory qualities (color, flavor, cutability, texture, appearance, and overall acceptability) of cakes were investigated. Significant regression models that explained the effects of different amounts of flour on all response variables were determined. The proposed model in this study had high $R^{2}$ and $R^{2}$ (adj). Therefore, the model was approved for fitting information. Finally, a gluten‐free sponge cake recipe was formulated using 1 g of oleaster flour, 1 g of pumpkin, and 10 g of quinoa flour to achieve the desired sensory quality.

## Graphical abstract

This research aimed to optimize the formulation of a sponge cake by adding quinoa, oleaster, and pumpkin flour. After completing the replacement, the resulting product is a gluten‐free cake suitable for celiac patients. Then, this study aimed to evaluate the impact of substitution on the physicochemical and sensory properties of gluten‐free sponge cakes using a mixture design methodology.

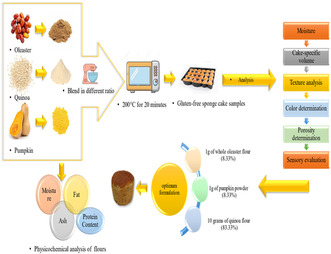

## 1 INTRODUCTION

Bakery products, such as cakes, are widely consumed and important for daily nutrition. Meanwhile, people with celiac disease, the most common food intolerance disorder, are sensitive to the gluten in the formulation of these products. Gluten replacement in gluten‐free products is one of the most challenging issues for cereal technologists. Numerous gluten‐free baking products are available on the market today, but unfortunately, many have low nutritional value (Bajerska et al., 2015; Gómez et al., 2008).

Pseudocereal quinoa (*Chenopodium quinoa* Willd.) is a dicotyledonous plant with a high nutritional content and traits that withstand stress, including resistance to drought, salt, and cold. Also, having properties such as mouthfeel, good texture, and functional characteristics is a good reason to use this flour as an enrichment and alternative to wheat flour in various products for celiac patients (Navruz‐Varli & Sanlier, 2016; Nowak et al., 2016).

Oleaster (*Elaeagnus Angustifolia*, Russian olive) belongs to the *Elaeagnus* L. genus and Elaeagnaceae family and can grow in different climates. Because of its high content of minerals, dietary fiber, phenolic compound, floury structure, special taste, and also improving the rheological properties of the batter, this ingredient can serve a functional purpose in the creation of baked goods. Adding oleaster to the cake formulation reduces hardness and stickiness and increases nutrition (Ayaz & Bertoft, 2001; Sahan et al., 2013).

Pumpkin is a seasonal crop belonging to the genus *Cucurbita moschata* of the family Cucurbitae that extensively grows in all tropical and subtropical countries. This product contains high amounts of dietary fiber, including pectin, which adds to bakery products and improves their quality and texture. Adding dietary fiber to bakery products has various technological functions, including the role of fat binder, gel binder, and texture improvement, which aids in enhancing the size and texture of the cake by increasing its volume and making it softer. Pumpkin powder has nutritional and functional properties, making it a versatile ingredient for baked goods and a natural coloring and flavoring agent (Abdul‐Hamid & Luan, 2000; de Carvalho et al., 2012).

Due to the need of society for such products with desirable nutritional and quality properties and acceptance by celiac patients, still trying to provide a new formulation in this field continues.

This study aimed to optimize the formulation of a sponge cake by adding quinoa, oleaster, and pumpkin flour. After completing the replacement, the resulting product is a gluten‐free cake suitable for celiac patients. Then, this study aimed to evaluate the impact of substitution on the physicochemical and sensory properties of gluten‐free sponge cakes using a mixture design methodology.

## 2 MATERIALS AND METHODS

### 2.1 Materials

Quinoa flour without saponin (Zidaasht Co., Iran) and whole oleaster powder were prepared from a grocery store located in Mashhad. Other materials used in this study, including fresh eggs (Telavang Co., Iran), sugar, baking powder, and vanilla, were purchased from a local market in Iran and then transferred to the laboratory. To be used in the formulation of cakes, each type of flour was kept in a plastic bag.

## 3 METHODS

### 3.1 Pumpkin powder production

Fresh pumpkins were processed into pumpkin powder by removing the peels, fibrous material, and seeds from *Cucurbita moschata*, whose flesh was cut into small pieces 5 mm thick. After blanching for 5 min at 98°C in boiling water, the pumpkin pieces were cooled to retain their flavor and aroma. After that, the blanched pieces of pumpkin were dried in a dryer at 60°C for 8 h, then powdered by a domestic grinder, and then passed through an 85 BSS sieve to get a uniform particle size. The flour was packed to prevent moisture exchange and stored at room temperature for later use (Bhat & Bhat, 2013; Pongjanta et al., 2006).

### 3.2 Preparation of cake

The egg whites were separated from the yolks and beaten with an electric mixer (Moulinex, France) on high speed for 3 min until they turned into a foam shape. The yolk was mixed with vanilla and sugar for 10 min with a mixer until it became a yellow cream. To make the dough, water was added to the yolk and stirred for 2 more minutes. The next step was to slowly add the flour and baking powder and gently mix them with a hand mixer until a consistent dough was formed. Once the dough was ready, it was poured into a paper mold of equal weight and baked in an oven (Daatis, Iran) at 200°C for 20 min. The gluten‐free sponge cakes were cooled, packaged in insulated bags, and stored at room temperature before analysis. Table 1 provides the formula for the control cake (cc), which includes constant basic components like sugar, egg, baking powder, vanilla, and water. Quinoa, oleaster, and pumpkin flour were used in the formula to create a gluten‐free sponge cake. To assess the impact of each flour on the cake's quality parameters, a mixture experimental design was utilized and the results are presented in Table 2.

**TABLE 1:** Control cake formula.

Ingredients	Amount (g)	% based on flour weigh	%
Wheat flour	12	100	21.35
Sugar	12	100	21.35
Egg	25	208.33	44.48
Baking powder	1	8.33	1.78
Vanilla	0.1	1.67	0.36
Water	6	50	10.68

**TABLE 2:** Mixture composition in the gluten‐free sponge cake formulated with oleaster (*X*_1_), pumpkin (*X*_2_), and quinoa (*X*_3_) in a three‐component extreme vertices mixture design.

Run	*X*_1_ (g)	*X*_2_ (g)	*X*_3_ (g)	StdOrder	RunOrder	PtType	Blocks
1	1.50	2.50	8.00	11	1	0	1
2	1.00	4.00	7.00	3	2	1	1
3	2.00	1.00	9.00	2	3	1	1
4	1.00	1.00	10.00	1	4	1	1
5	1.50	2.50	8.00	5	5	0	1
6	1.50	2.50	8.00	10	6	0	1
7	1.75	1.75	8.50	7	7	−1	1
8	1.25	3.25	7.50	8	8	−1	1
9	1.25	1.75	9.00	6	9	−1	1
10	1.75	3.25	7.00	9	10	−1	1
11	2.00	4.00	6.00	4	11	1	1

### 3.3 Experimental design

A three‐extreme vertices mixture design was applied to optimize the proportions of three flours in gluten‐free sponge cake: oleaster (*X*_1_), pumpkin (*X*_2_), and quinoa (*X*_3_). The applied range of flour was 1 ≤ oleaster ≤ 2, 1 ≤ pumpkin ≤ 4, and 6 ≤ quinoa ≤ 10 g. The plan was created using mixture design software version 7, as shown in Table 2. Then the optimal formulation was obtained based on sensory analysis as a response factor. Component proportions were expressed as fractions of the mixture with a sum (*X*_1_ + *X*_2_ + *X*_3_) equal to 12 g (Equation 1) (refer to a basic formulation for each cupcake).

### 3.4 Statistical and data analysis

Design‐Expert software (version 7) was used for experimental design and analysis. Linear models (Equations 2) were fitted to the experimental data in this study.

(2) $$\;Y=\beta_{1}X_{1}+\beta_{2}X_{2}+\beta_{3}X_{3}$$

where *Y* is the dependent variable that is predicted, and *β*_1_, *β*_2_, and *β*_3_ are the equation coefficients. The effect of variables on responses was examined using analysis of variance (ANOVA). Additionally, the model's adequacy was checked by calculating lack of fit, ($R^{2}$), adj‐$R^{2}$, CV, and PRESS.

### 3.5 Physicochemical analysis of flours

Moisture (AACC method 44‐16), ash (AACC method 08‐01), fat (AACC method 10‐30), and protein contents (AACC method 46‐12) of flour samples were analyzed according to AACC (The American Association of Cereal Chemists Methods) (1995).

### 3.6 Physicochemical characterization of gluten‐free sponge cake

The moisture content of cakes was determined using standard methods (AOAC, 1990).

Cake‐specific volume was calculated as the ratio between the cake volume and its weight (mL/g) according to AACC Method 72‐10 (AACC, 2000).

### 3.7 Texture analysis of gluten‐free sponge cake

To determine the texture of cake samples, the textural profile analysis (TPA) test was used. A slice of cake measuring 2 × 2 × 2 cm from the middle section was selected for the test. The texture analyzer (TA plus Lioyd, USA) was used to measure textural parameters such as hardness, gumminess, cohesiveness, chewiness, and springiness. A 45 mm diameter cylindrical probe compressed the cake to 50% at a speed of 50 mm/min and applied a force of 100 N. All measurements were taken in a controlled room at 25°C, 2 h after baking (Gularte et al., 2012).

### 3.8 Color determination of gluten‐free sponge cake

The image processing technique was applied to determine the color of both the crust and crumb of the samples. Cake crust color analysis was conducted 2 h after baking and involved three indicators. The brightness of the sample was represented by the index L*, which ranged from zero (pure black) to 100 (pure white). The index a* reflected the degree of similarity of the sample color to green and red, ranging from −120 (pure green) to +120 (pure red). Meanwhile, the index b* represented the degree of similarity of the sample color to blue and yellow, ranging from −120 (pure blue) to +120 (pure yellow). To measure these indicators, a 2 × 2 cm portion of the cake was first prepared and photographed using a Nikon 1300D camera with a 300‐pixel resolution. The resulting images were then transferred to Image J software, where the LAB space in the Plugins section was activated to calculate the aforementioned indicators (Du & Sun, 2004; Naji‐Tabasi & Mohebbi, 2015).

### 3.9 Porosity determination of gluten‐free sponge cake

To evaluate the porosity of the cake samples, an image processing technique was used 2 h after baking. Slices were obtained from the middle part of the cake and placed in a room with black walls that prevented light reflection. The Image J software was used to analyze the prepared image. A 500 × 500 pixel piece was isolated from the images, and a gray surface image was created by activating the 8‐bit part of Image J software. To convert the gray images to binary images, the images were first contrast‐enhanced and then thresholded. The binary part of the software was then activated, resulting in dark and light spots. The number of cavities in the cake was proportional to the ratio of light to dark spots. By activating the software analysis section, this ratio was calculated to measure the porosity of the cake samples (Shahidi et al., 2011).

### 3.10 Sensory evaluation of gluten‐free sponge cake

Fourteen individuals aged 18–60, both male and female, conducted sensory evaluations of the samples using the hedonic scale method. Each sample was assigned a three‐digit random code and randomly presented to the panel. The panelists rated each sample on a 9‐point hedonic scale based on color, flavor, appearance, cutability, and texture. The scale ranged from 1 (dislike extremely) to 9 (like extremely) (de Souza et al., 2018). Overall acceptability was calculated based on the average of total points. To minimize any remaining effects, the evaluators were given water to wash their mouths between different samples.

## 4 RESULTS AND DISCUSSION

The analysis of variance data for response variables along with the correlation coefficient is given in Tables 4, 5, 6, 7. To determine the adequacy of models, lack‐of‐fit, model analysis, and $R^{2}$ were examined. The lack‐of‐fit measures the ability of a model to represent the data in the experimental domain and should be insignificant in an inadequate model. The aptness of the model to signify a real relationship among selected parameters is given by $R^{2}$. The $R^{2}$ values of the models for this study were >.70. In a fit model, a coefficient of determination of more than 80% is acceptable, but some researchers have accepted a coefficient of determination of more than 70% (Jan et al., 2018). The results indicate that there is no significant lack of fit in all the response variables at satisfactory levels of $R^{2}$ of more than 70%.

### 4.1 Chemical properties of flours

Table 3 shows the chemical composition of the flours. Quinoa flour showed the maximum protein content (13.3%). The whole oleaster flour had the highest moisture content (11.271%) due to its high fiber content. According to the previous literature, fibers specifically contribute to the hydration properties, which cause an increase in water interactions through hydrogen bonding (Zanganeh et al., 2020).

**TABLE 3:** Proximate composition of flours.

Parameters	Wheat flour	Quinoa flour	Oleaster powder	Pumpkin powder
Moisture (%)	12.195	7.178	11.271	8.207
Total ash (%)	0.6345	2.374	1.932	8.406
Total fat (%)	2.055	7.178	8.20	8.720
Total protein (%)	9.505	13.30	5.73	10.40

### 4.2 Physicochemical characterization of gluten‐free sponge cake

#### 4.2.1 Moisture

**FIGURE 1:**
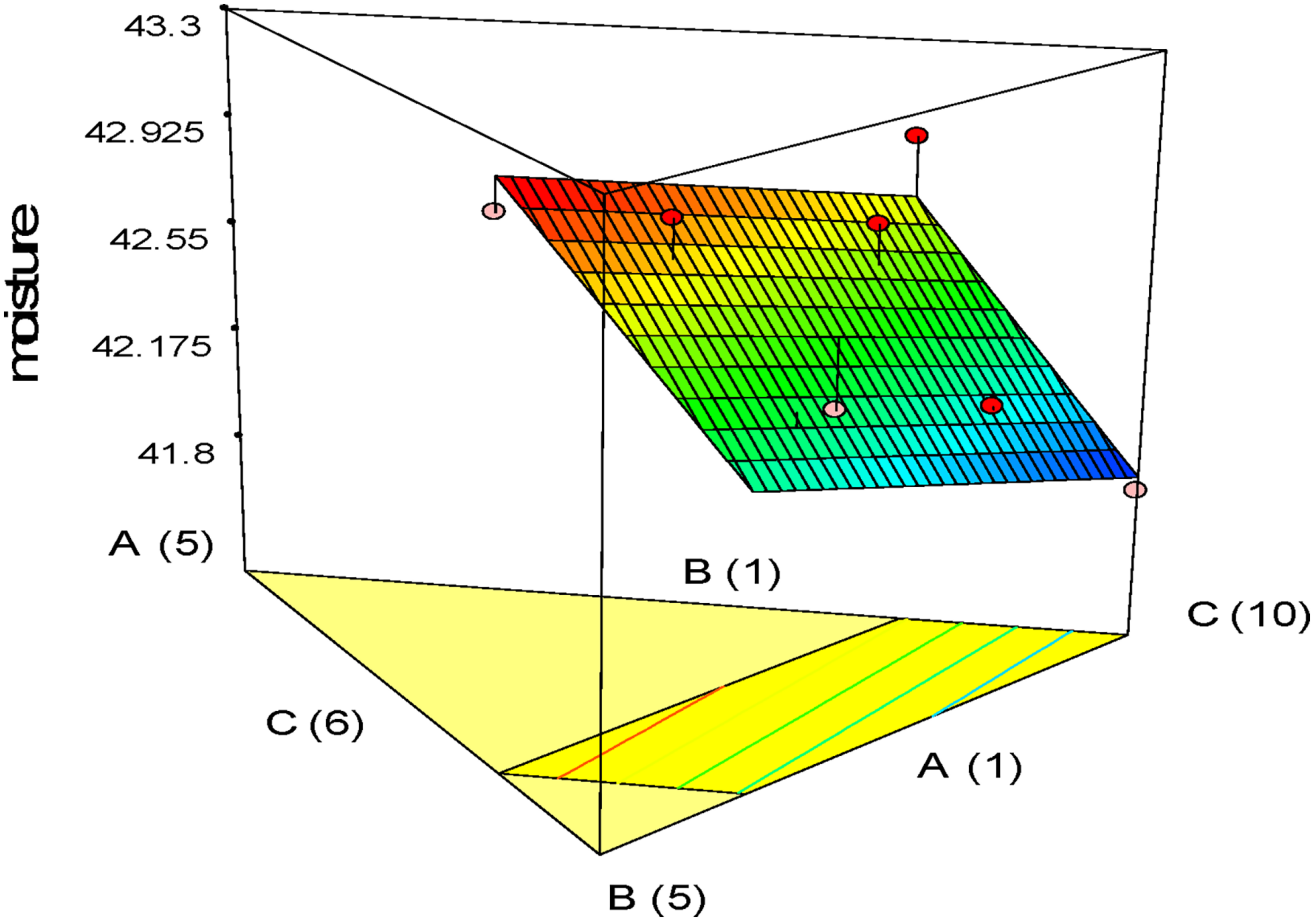
Response plots showing the effect of variables on the moisture level of gluten‐free sponge cake samples.

Table 4 shows the results of the physicochemical characterization of gluten‐free cake. According to the results, the fitted linear model for moisture values was statistically significant (*p* < .05), but the lack of fit index was not significant. Based on Figure 1, the effect of adding whole oleaster flour and then pumpkin powder on increasing the moisture content was greater than that of quinoa flour. Adding fiber sources to the cake, such as apple pomace, increases the water absorption capacity of the flour (Sudha et al., 2007). Also, the fiber obtained from fruits, which contain higher pectin, has a higher water‐holding capacity than the fibers of cereals and legumes (Chen et al., 1988). Glucose and fructose have strong hydrophilicity and high solubility. The high affinity of the pumpkin fibrous matrix for water molecules provides conditions for more glucose molecules to establish hydrogen bonds with water. This causes hydrogen bonds to increase and free water mobility to decrease, thereby increasing cake moisture (de Escalada Pla et al., 2007).

**TABLE 4:** Regression coefficients and analysis of variance of gluten‐free sponge cake characteristics.

Source	Moisture%	Specific volume (mL/g)
*β*_1_	4.3184****	0.4394****
*β*_2_	3.5386***	0.1533***
*β*_3_	3.4007**	0.2086**
Model (*p*‐value)	0.0024**	0.0037***
Lack of fit (*p*‐value)	4858/0^ns^	1258/0^ns^
*R* ^2^	7780/0	7527/0
Adj‐*R* ^2^	7225/0	6909/0
CV (%)	50/0	37/2
Press	86/0	064/0

*Note*: ^ns^, *, **, ***, **** respectively, they indicate non‐significance, significance at *p* < 0.05 level, significance at *p* < 0.01 level, significance at *p* < 0.001 level and significance at *p* < 0.0001 level.

The study conducted by Ptitchkina et al. (1998) also indicated that incorporating pumpkin powder into bread resulted in higher moisture levels because fiber can form hydrogen bonds with water molecules in the formulation and increase the moisture content of the product due to its hydroxyl groups. In a study by Zanganeh et al. (2020), the addition of oleaster flour to gluten‐free cake resulted in a higher moisture content compared to the control sample. The results of Jaldani et al. (2017) also confirmed that the increase in the moisture content of the samples with the increase in the level of quinoa flour is due to the large number of hydroxyl groups in its structure, which causes the absorption and retention of water molecules in the product during cooking.

#### 4.2.2 Specific volume

The specific volume of a cake is a crucial factor in determining its appeal to consumers. During the baking process, the cake dough measures the amount of air, water vapor, and carbon dioxide produced and the changes that occur (Zanganeh et al., 2020).

The specific volume of the CC produced by wheat flour was determined to be 4.65 mL/g. The specific volume of the treatments varied between 2.51 and 2.89 mL/g.

Based on the results of ANOVA, the fitted linear model was statistically significant (*p* < .05) for specific volume values, but the lack of fit index was not significant. Oleaster, quinoa, and pumpkin flour showed a significant linear effect (Table 4).

**FIGURE 2:**
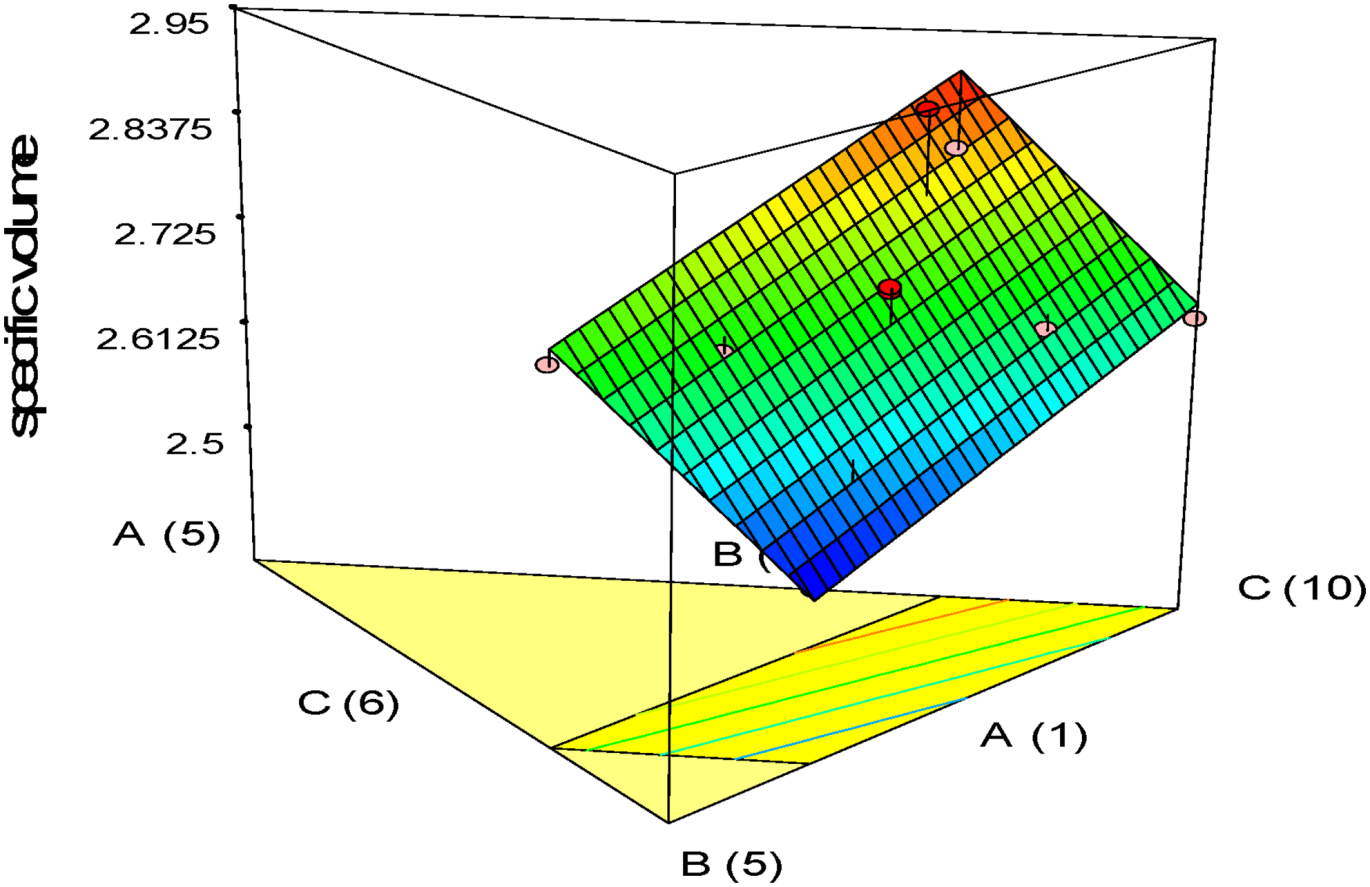
Response plots showing the effect of variables on a specific volume of gluten‐free sponge cake samples.

The results displayed in Figure 2 illustrate the impact of independent variables on sample volume. Increasing the amount of whole oleaster flour caused an increase in specific volume. Using higher amounts of quinoa resulted in decreased specific volume, while lower amounts led to an increase. Due to the high protein content of quinoa flour, which improves dough viscoelasticity and increases protein‐starch interaction, the cake's specific volume increased significantly when the amount of flour was increased (Bozdogan et al., 2019). Alencar et al. (2015) reported that adding quinoa flour to gluten‐free bread increases the specific volume of samples.

The specific volume of the samples decreased as the amount of pumpkin powder increased. The cake's volume is reduced when fiber sources are added because they can block the water in the cake's structure. This reduction in free water content prevents the gas bubbles from collapsing during the baking stage (Gularte et al., 2012). The presence of fiber in the cake formula and high water absorption reduce its specific volume. This is because increasing water absorption reduces the dough's viscosity, which, in turn, reduces its gas‐holding capacity and, ultimately, its specific volume (Yildiz & Dogan, 2014).

Singh et al. (2016) found that incorporating carrot fiber into gluten‐free cake decreased specific volume by collapsing CO2 bubbles during baking. The specific volume of the cake increases due to the strong hydrophilicity of apple fiber (Chen et al., 1988).

According to Gallagher et al. (2004), gluten, the primary protein in wheat flour, plays a crucial role in creating a viscoelastic network that helps trap gas in baked goods. Without this network, the volume of gluten‐free cake decreases.

### 4.3 Texture analysis of gluten‐free sponge cake

#### 4.3.1 Hardness

**FIGURE 3:**
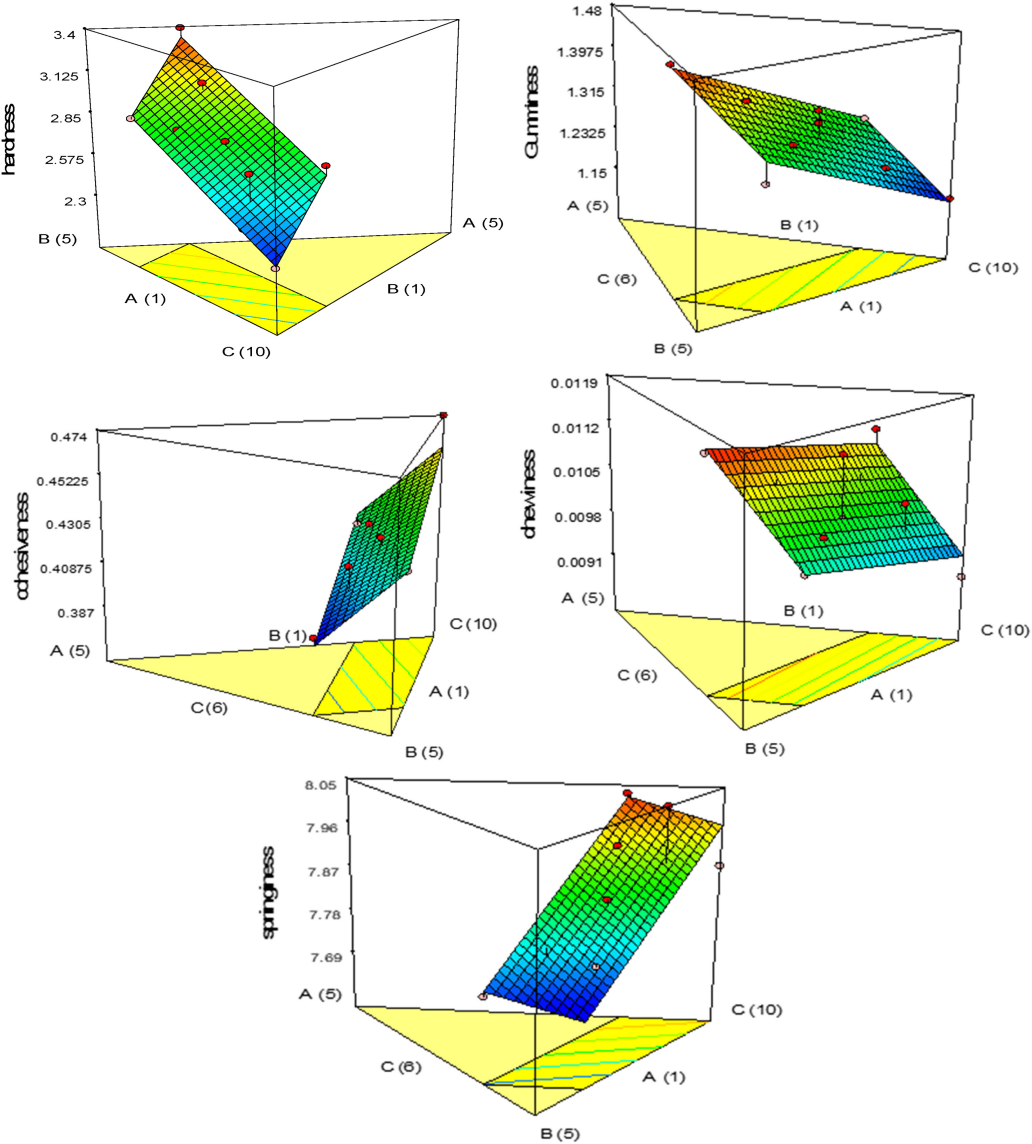
Response plots showing the effect of variables on the texture of gluten‐free sponge cake samples.

In terms of sensory perception, hardness is defined as the amount of force needed to deform food when bitten (Esteller et al., 2004). Between 2.345 and 3.390 N were the hardness values of the cake made with quinoa, oleaster, and pumpkin flour. The TPA results in Table 5 demonstrated that all linear terms had a significant effect (*p* < .05) on the cakes' hardness, with the *p*‐value indicating this. As the quinoa flour increased, the hardness of the sample decreased, consistent with the results of Bozdogan et al. (2019). As the amount of whole oleaster flour increased, the hardness increased (Figure 3). Zanganeh et al. (2020) showed that by increasing the percentage of whole oleaster flour, the perception of hardness increased. In gluten‐free products, due to the lack of a gluten network, moisture migrates from the crumb to the cake crust, and the main reason for the hardening of the cake texture can be the crystallization of starch components, especially amylopectin, during cake storage. Other studies have reported that the addition of dietary fiber can increase the hardness of cereal products. Based on the coefficients of the model components, pumpkin powder had the greatest effect on increasing the hardness of the samples, followed by oleaster flour (Chen et al., 1988; Gallagher et al., 2004). The fiber‐protein complex at high cake baking temperatures can also cause the hardening of the final product (Jan et al., 2018).

**TABLE 5:** Regression coefficients and analysis of variance of texture characteristics of gluten‐free sponge cake

Source	Springiness	Chewiness	Cohesiveness	Gumminess	Hardness	Porosity
*β*_1_	0.7191****	2.2551****	9.9429****	0.2257****	0.5494****	8.6646****
*β*_2_	0.5763***	8.2422***	0.0302***	0.1330***	0.3378***	2.1606***
*β*_3_	0.6681***	6.4235**	0.0418**	0.0796**	0.1471**	1.3102**
Model (*p*‐value)	001/0***	0.003***	0022/0**	0.0005****	0.0002****	0001/0^****^
Lack of fit (*p*‐value)	1400/0^ns^	8825/0^ns^	3414/0^ns^	2742/0^ns^	3936/0^ns^	9752/0^ns^
*R* ^2^	8193/0	7667/0	7836/0	8519/0	8814/0	9793/0
Adj‐*R* ^2^	7741/0	7084/0	7295/0	8149/0	8517/0	9742/0
CV (%)	66/0	51/3	31/2	73/2	56/3	5/1
Press	049/0	9/1	8/1	019/0	13/0	10/2

*Note*: ^ns^, *, **, ***, **** respectively, they indicate non‐significance, significance at *p* < 0.05 level, significance at *p* < 0.01 level, significance at *p* < 0.001 level and significance at *p* < 0.0001 level.

#### 4.3.2 Gumminess

Gumminess refers to the amount of energy required to chew solid food until it reaches an appropriate texture for swallowing (Razavi & Akbari, 2013). The gumminess values of the cohesiveness of gluten‐free sponge cakes changed between 1.160 and 1.467 N. The fitted linear model was statistically significant (*p* < .05) according to Table 5's analysis of variance results, but the model's lack of fit index was not significant. Oleaster flour, quinoa, and pumpkin illustrated a significant linear effect. At medium and high rates of oleaster, gumminess increased, which is consistent with the findings of Zanganeh et al. (2020). With the increase in pumpkin powder, gumminess decreased; furthermore, gumminess decreased significantly with the addition of quinoa flour (Figure 3).

#### 4.3.3 Cohesiveness

The cohesiveness values of gluten‐free sponge cakes were found to range from 0.395 to 0.473. Cohesiveness is a measure of the food structure's ability to resist compression and is a critical factor in creating high‐quality products that meet consumer and packaging standards (Bozdogan et al., 2019). Also, the low consistency of the cake has a negative effect on its acceptance by the consumer and indicates the possibility of improper fragmentation and high disintegration (Masmoudi et al., 2020). After analyzing variance (Table 5), the fitted linear model for continuity values was found to be statistically significant (*p* < .05), but the lack of fit index was not significant. Oleaster, quinoa, and pumpkin flour showed a significant linear effect (*p* < .05). According to Zanganeh et al. (2020), high amounts of oleaster flour decreased the degree of cohesiveness. But with the increase in pumpkin powder, the consistency increased. Quinoa flour at medium and high levels increased the cohesiveness (Figure 3). The cake formula compounds' ability to absorb and retain water increases cohesiveness (Jaldani et al., 2017).

#### 4.3.4 Chewiness

The chewiness values for gluten‐free sponge cakes ranged from 0.0091 to 0.0115. Sensory evaluation showed that chewiness refers to the amount of energy needed to grind semi‐solid food until it can be swallowed (Razavi & Akbari, 2013). According to the results of the analysis of variance (Table 5), the fitted linear model for chewiness exhibited statistical significance (*p* < .05), but the lack of fit index for this model was not significant. Oleaster flour, quinoa, and pumpkin showed a significant linear implication. Based on the three‐dimensional diagram in Figure 3, oleaster flour increased chewiness at high and medium levels, but this index decreased with the decrease of oleaster flour and the increase of pumpkin powder. Chewiness also decreased with the increase in quinoa flour. Majzoobi et al. (2016) discovered that the chewiness of a gluten‐free cake was enhanced by adding carrot powder.

#### 4.3.5 Springiness

The springiness value of the treatments varied between 7.715 and 8.033 mm. From a sensory point of view, the return value of the deformed material to its original condition (without deformation) after removing the chewing force is called elasticity (Razavi & Akbari, 2013). The elasticity of the cake core is related to protein accumulation and indicates the freshness and elasticity of the product (Shevkani & Singh, 2014). Based on the outcomes of the ANOVA in Table 5, the fitted linear model for springiness was statistically significant (*p* < .05), and the lack of fit index was not significant. Quinoa, oleaster, and pumpkin flour showed a significant linear effect. Figure 3 shows that as the amount of quinoa and oleaster flour increased, the elasticity of the cake samples increased as well. However, the elasticity decreased as the level of pumpkin powder increased, likely due to the presence of fibrous compounds in the pumpkin. The elasticity of gluten‐free cakes increases with the addition of quinoa flour, which indicates that quinoa flour improves the elasticity of gluten‐free cakes because it has a high protein content (Bozdogan et al., 2019).

### 4.4 Color determination of gluten‐free sponge cake

#### 4.4.1 The crust color of gluten‐free sponge cake

The color of food is a key quality factor. It is influenced by the formulation and baking processes of bakery and confectionery products. The color of a product influences its acceptance by consumers, as it is the first factor in food evaluation and consumption (da Silva et al., 2009). The color indexes (a*, b*, L*) of gluten‐free cake crust and crumb are given in Table 6.

**TABLE 6:** Regression coefficients and analysis of variance of the colorimetric characteristics of crust and crumb of gluten‐free sponge cake.

Source	crumb			crust		
	b*	a*	L*	b*	a*	L*
*β*_1_	1.1267****	0.8898****	−2.8515****	1.2396****	0.3963****	1.0264****
*β*_2_	4.7574***	−0.1198 ^ns^	3.9029***	3.5963***	1.7531***	2.5392***
*β*_3_	3.1040**	−0.8961***	5.2250***	1.3917**	0.9638**	1.3109**
Model (*p*‐value)	0001/0^****^	0001/0^****^	0.0011**	0001/0^****^	0.004**	0001/0^****^
Lack of fit (*p*‐value)	3806/0 ^ns^	^ns^ 5410/0	^ns^ 5667/0	^ns^ 2728/0	4375/0 ^ns^	2851/0 ^ns^
*R* ^2^	9060/0	8981/0	8195/0	9524/0	8625/0	9080/0
Adj‐*R* ^2^	8825/0	8726/0	7744/0	9405/0	8282/0	8850/0
CV (%)	77/1	37/6	53/3	66/2	03/3	53/2
Press	31/8	64/2	15/3	73/6	35/2	83/3

*Note*: ^ns^, *, **, ***, **** respectively, they indicate non‐significance, significance at *p* < 0.05 level, significance at *p* < 0.01 level, significance at *p* < 0.001 level and significance at *p* < 0.0001 level.

**FIGURE 4:**
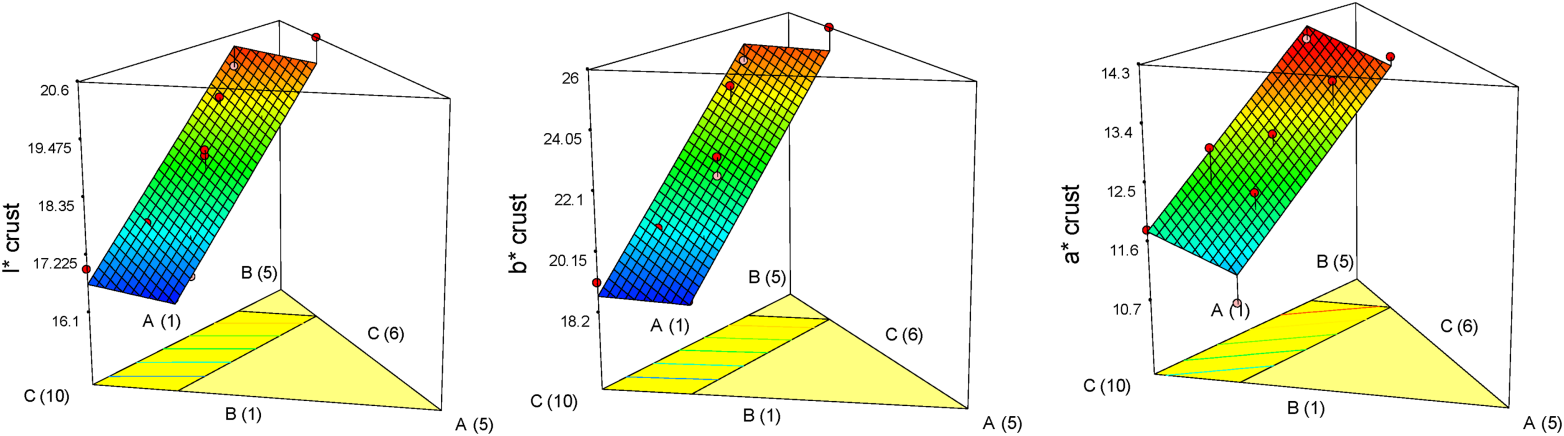
Response plots showing the effect of variables on the amount of crust color of gluten‐free sponge cake samples.

Based on the results of ANOVA, the fitted linear model for a*, b*, and L* was statistically significant (*p* < .05), but the lack of fit index was not significant. The Maillard reaction between the sugar and amine groups and the caramelization of sugars contribute to the color development of the cake shell during baking, though the color of the ingredients in the cake recipe can also have an impact (Paesani et al., 2021). The linear effects of oleaster, quinoa, and pumpkin flour were significant for a*, b*, and L* indexes of gluten‐free cake crust color. Figure 4 displays the impact of independent variables on these indicators. According to the coefficients of the model components, pumpkin powder at high levels increased the b* index and enhanced the cake crust's yellow color, however, oleaster flour and quinoa flour had less of an impact. Based on the results of Jaldani et al. (2017), the b* index was not significantly affected by adding quinoa flour to the gluten‐free cake.

According to Figure 4, high levels of pumpkin powder had the greatest effect on increasing the a* index. This result is consistent with the results of Sudha et al. (2007). Also, quinoa flour at high levels and oleaster flour at medium levels increased the redness of the gluten‐free cake crust. The a* index of the crumb and crust, which shows the degree of redness, increases due to the rise in quinoa flour. The Maillard reaction and caramelization are responsible for the cake crust's increased red color. The protein content increase in the cake intensifies the Maillard reaction and creates a darker color due to the additional quinoa flour (Bozdogan et al., 2019). The results of Zanganeh et al. (2020) also confirmed that the addition of oleaster flour reduces the lightness of the cake crust and makes the cake crust darker than the control sample. All variables had a significant effect on reducing the brightness of the cakes; however, as shown in Figure 4, quinoa flour and oleaster flour had a greater role in reducing the brightness of the cakes' crust than pumpkin powder.

#### 4.4.2 The crumb color of gluten‐free sponge cake

**FIGURE 5:**
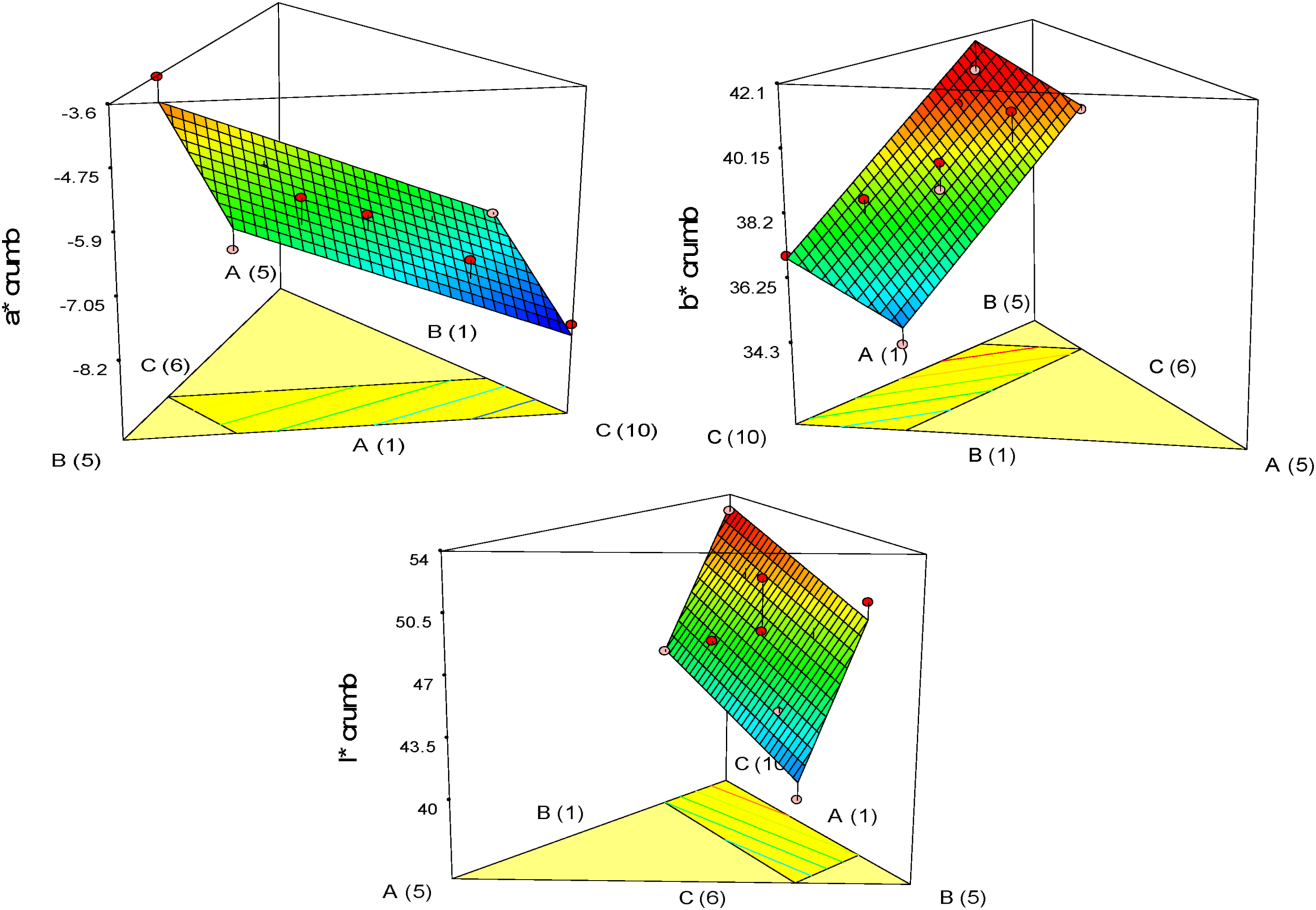
Response plots showing the effect of variables on the amount of crumb color of gluten‐free sponge cake samples.

Table 6 presents the findings of the variance analysis. The results indicate that the a*, b*, and L* indexes had statistical significance (*p* < .05) and that there was no significant lack of fit in any of the response variables. All three variables had a significant linear effect on the reduction of the a*, b*, and L* indexes of cake crumb. Considering Figure 5, the role of pumpkin powder in increasing the yellowness of the cake crumb is greater. By increasing the level of quinoa flour, the amount of yellowness of the cake crumb increases, but by increasing the amount of oleaster flour, the amount of b* factor decreases. Considering that the baking temperature is higher than 100°C, the color of the ingredients of the cake is affected by the Maillard reaction, and as a result, the color of the crumb changes (Paesani et al., 2021). Adding flour from pseudocereals like buckwheat, amaranth, and quinoa to gluten‐free bread increased the bread crumb's yellow color compared to control samples (Alencar et al., 2015). The color of the crust and crumb of the gluten‐free cake significantly decreased when apple puree was added, as shown by Sudha et al. (2007).

### 4.5 Porosity of gluten‐free sponge cakes

**FIGURE 6:**
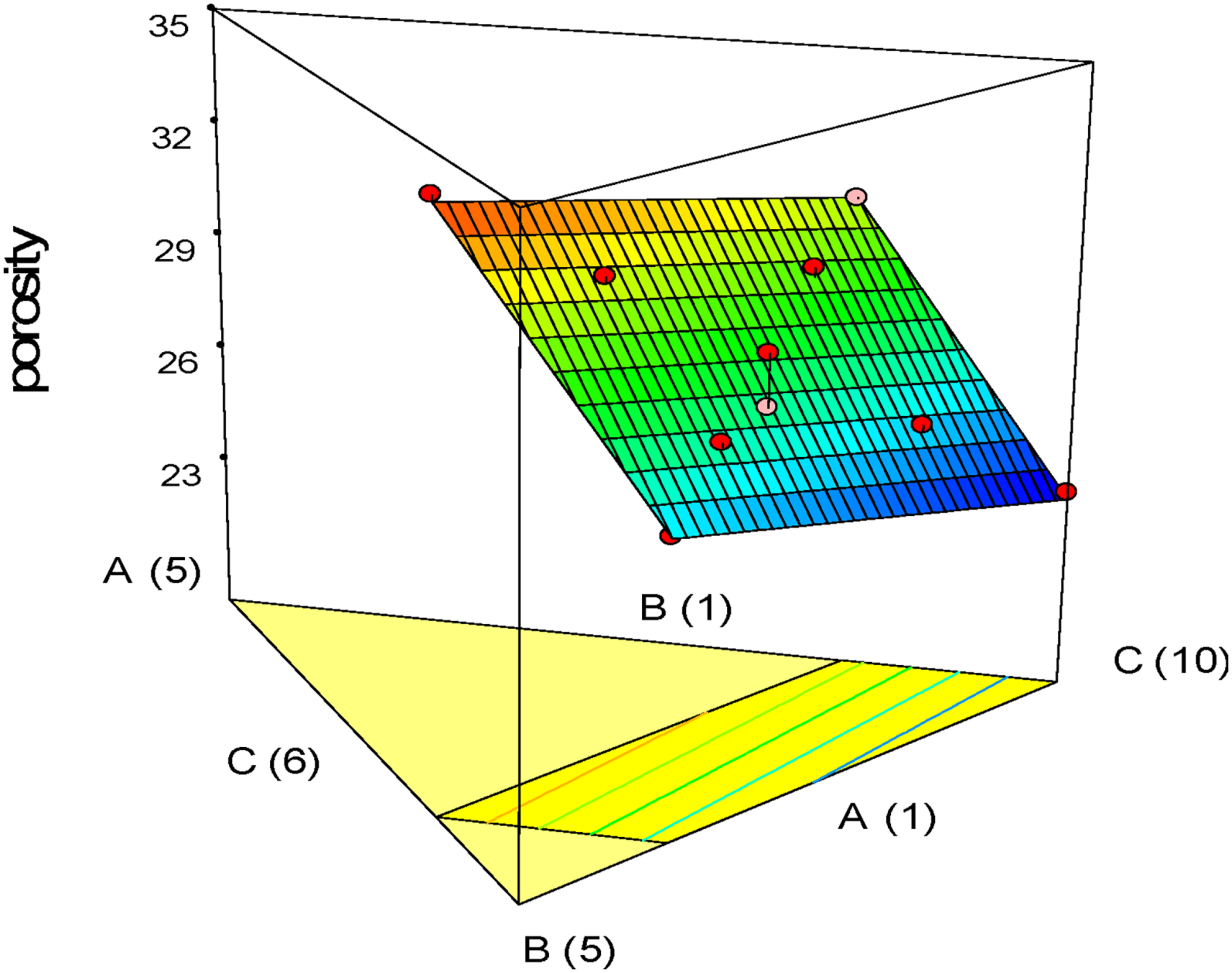
Response plots showing the effect of variables on the porosity of gluten‐free sponge cake samples.

Porosity affects cake quality and is determined by the quantity and arrangement of holes. Increasing the number of gas‐filled cavities and distributing them evenly enhances porosity (Saeidi et al., 2018). The porosity of gluten‐free sponge cake samples was measured and found to range between 24.0858% and 32.9912%, with the control sample measuring 23.1186%. The analysis of the variance results is shown in Table 5. It was discovered that the porosity‐fitted linear model was statistically significant (*p* < .05); however, the lack of fit index was not significant. The linear effect of oleaster, quinoa, and pumpkin powder was also significant. Based on the three‐dimensional diagrams in Figure 6, oleaster flour increased the porosity of samples in the upper and middle levels, while pumpkin powder reduced the porosity and made the internal cavities uniform. Quinoa decreased porosity at high levels but increased it at medium and lower levels. Studies by Jaldani et al. (2017) have confirmed that increasing the quantity of quinoa flour in gluten‐free cakes results in a significant reduction in porosity. Similarly, Huang and Yang (2019) found that adding red seaweed powder, which is high in fiber and water absorption, to cakes results in smaller inner cavities. On the other hand, incorporating quinoa flour in formulations of gluten‐free bread led to a structure with larger pores (Alencar et al., 2015). Pumpkin powder enhances the stability of gas cells by strengthening the matrix around them due to the surface activity of pumpkin pectin, preventing further expansion of air inside the cavities (Gómez et al., 2007; Ptitchkina et al., 1998). However, when added to cakes, pumpkin powder makes the holes smaller and more uniform (Vatandoust et al., 2015; Zamordi et al., 2018).

### 4.6 Sensory evaluation of gluten‐free sponge cakes

**FIGURE 7:**
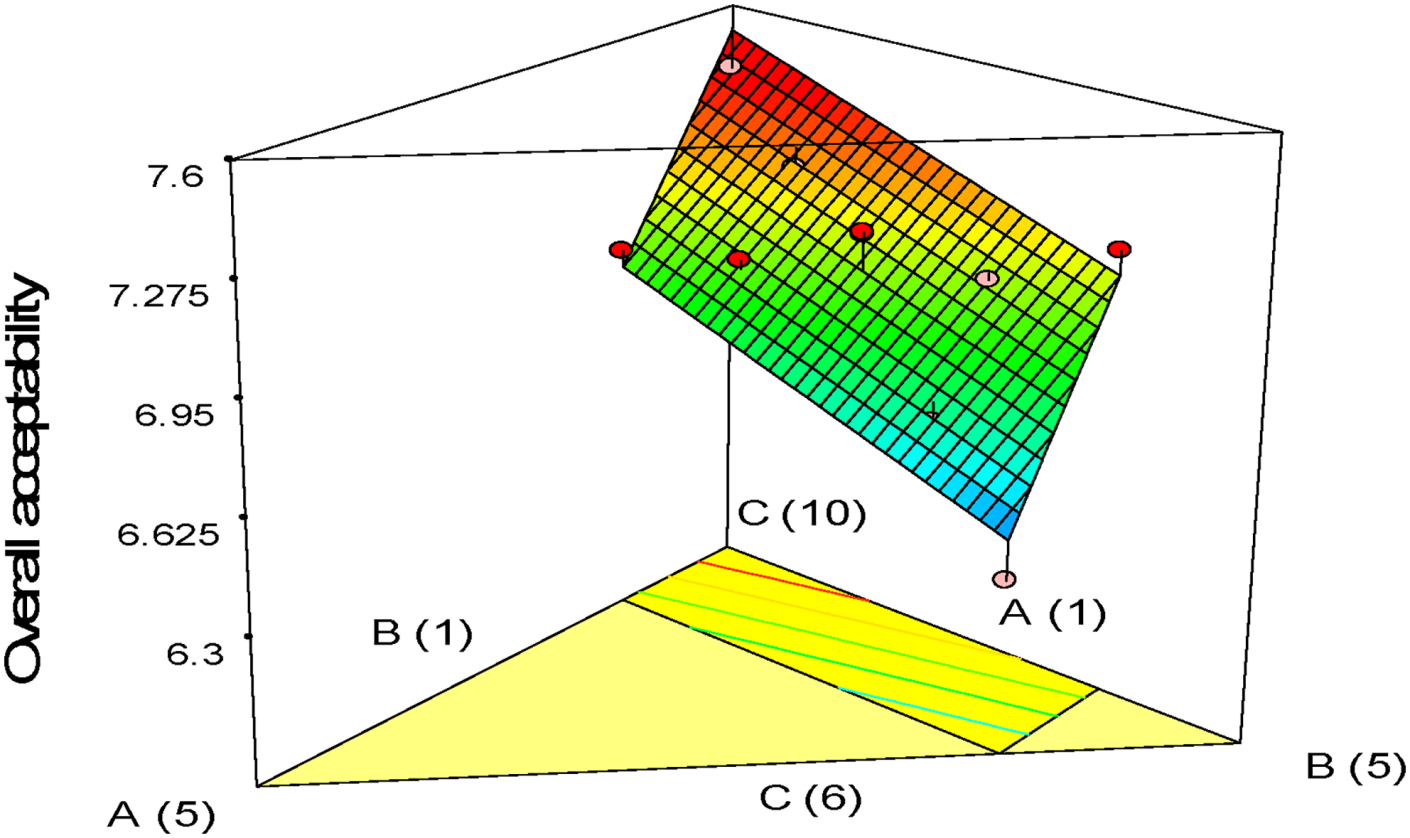
Response plots showing the effect of variables on the overall acceptability of gluten‐free sponge cake samples.

The sensory analysis results for gluten‐free sponge cakes are exhibited in Table 7. According to the ANOVA findings, the fitted linear model was significant (*p* < .05) for all factors, but the lack of fit index was not significant. According to Figure 7, the addition of more quinoa flour to treatments increased overall acceptance. The reason for this is that with the increase in the level of quinoa flour, the texture, appearance, volume, and flavor of the cake samples were more acceptable to the sensory evaluators. Also, after quinoa flour, the effect of pumpkin powder on the overall acceptance rate of the samples was higher. Adding pumpkin powder had a great masking effect on the bitter taste of quinoa because of its special sweet taste. Adding pumpkin powder to bakery products enhances their taste and color, making them more appealing to consumers. This is supported by sensory acceptance studies (Ptitchkina et al., 1998). However, using whole oleaster flour in cake recipes can lead to reduced sensory acceptance due to the darkening of cake samples, volume reduction, and negative effects on texture. This is because the denser cake structure is less palatable (Sudha et al., 2007). Interestingly, combining these three flours could be a suitable alternative to wheat flour in cake recipes. Moreover, these flours have useful functional and nutritional properties, making them a great snack option for those who have celiac disease.

**TABLE 7:** Regression coefficients and variance analysis of gluten‐free sponge cake sensory evaluation test.

Source	Overall acceptability	Color	Flavor	Cutability	texture	Appearance
*β*_1_	0.0846****	−0.0646****	0.4336****	−0.0116****	0.2016****	−0.0234****
*β*_2_	0.5470***	0.4481***	0.4054***	0.5940***	0.6019***	0.5130***
*β*_3_	0.6894***	0.6996**	0.5557**	0.7812**	0.7174**	0.7788**
Model (*p*‐value)	0001/0^****^	0001/0^****^	0.0014***	0014/0**	0001/0^****^	0.0003****
Lack of fit (*p*‐value)	3105/0^ns^	0983/0^ns^	4359/0^ns^	1871/0^ns^	1881/0^ns^	6798/0^ns^
*R* ^2^	9110/0	9726/0	8061/0	8056/0	9002/0	8649/0
Adj‐*R* ^2^	8887/0	9657/0	7577/0	7570/0	8753/0	8311/0
CV (%)	30/1	08/1	48/1	44/2	08/1	36/2
Press	17/0	082/0	17/0	48/0	10/0	48/0

*Note*: ^ns^, *, **, ***, **** respectively, they indicate non‐significance, significance at *p* < 0.05 level, significance at *p* < 0.01 level, significance at *p* < 0.001 level and significance at *p* < 0.0001 level.

## 5 CONCLUSION

The current research focuses on optimizing gluten‐free sponge cake using quinoa, oleaster, and pumpkin flour with a mixture design approach. The statistical analysis shows that the models are adequate. The results indicate a significant correlation between variables and responses. Quinoa flour causes a decrease in moisture content and volume at higher levels while increasing specific volume at medium levels. It reduced hardness, gumminess, and chewability and increased cohesiveness, elasticity, and a* index of the crust. Similarly, oleaster flour and pumpkin powder increased moisture content. Oleaster flour increased hardness, gumminess, and porosity but decreased the cohesiveness and index b* of the cake crumb. High levels of pumpkin powder reduced specific volume, gumminess, chewability, elasticity, and porosity.

According to the overall results, the optimum formulation with the desired quality appreciated by the panelists could be obtained by incorporating 1 g of whole oleaster flour (8.33%), 1 g of pumpkin powder (8.33%), and 10 grams of quinoa flour (83.33%), making it a functional gluten‐free sponge cake for people with celiac and non‐celiac diseases.

## AUTHOR CONTRIBUTIONS

**Mahshad Madadi:** Conceptualization (equal); data curation (lead); formal analysis (lead); investigation (lead); methodology (equal); software (lead); visualization (equal); writing – original draft (lead); writing – review and editing (equal). **Sahar Roshanak:** Conceptualization (equal); methodology (equal); supervision (equal); writing – review and editing (equal). **Fakhri Shahidi:** Conceptualization (equal); funding acquisition (lead); project administration (equal); supervision (equal); validation (equal). **Mohammad Javad Varidi:** Resources (equal); supervision (equal).

## CONFLICT OF INTEREST STATEMENT

The authors declare no competing interests.

## ETHICS STATEMENT

This study does not involve any human or animal testing.

## Data Availability

All data generated or analyzed during this study are included in this published article.
